# Beneficial Microorganisms for Corals (BMC): Proposed Mechanisms for Coral Health and Resilience

**DOI:** 10.3389/fmicb.2017.00341

**Published:** 2017-03-07

**Authors:** Raquel S. Peixoto, Phillipe M. Rosado, Deborah Catharine de Assis Leite, Alexandre S. Rosado, David G. Bourne

**Affiliations:** ^1^Instituto de Microbiologia Paulo de Góes, Universidade Federal do Rio de JaneiroRio de Janeiro, Brazil; ^2^Instituto Museu Aquário Marinho do Rio de Janeiro-AquaRio (IMAM/AquaRio) – Rio de Janeiro Marine Aquarium Research CenterRio de Janeiro, Brazil; ^3^College of Science and Engineering, James Cook University, TownsvilleQLD, Australia; ^4^Australian Institute of Marine Science, TownsvilleQLD, Australia

**Keywords:** beneficial microorganisms for corals, BMC, probiotics, symbiosis, reversing dysbiosis

## Abstract

The symbiotic association between the coral animal and its endosymbiotic dinoflagellate partner *Symbiodinium* is central to the success of corals. However, an array of other microorganisms associated with coral (i.e., Bacteria, Archaea, Fungi, and viruses) have a complex and intricate role in maintaining homeostasis between corals and *Symbiodinium*. Corals are sensitive to shifts in the surrounding environmental conditions. One of the most widely reported responses of coral to stressful environmental conditions is bleaching. During this event, corals expel *Symbiodinium* cells from their gastrodermal tissues upon experiencing extended seawater temperatures above their thermal threshold. An array of other environmental stressors can also destabilize the coral microbiome, resulting in compromised health of the host, which may include disease and mortality in the worst scenario. However, the exact mechanisms by which the coral microbiome supports coral health and increases resilience are poorly understood. Earlier studies of coral microbiology proposed a coral probiotic hypothesis, wherein a dynamic relationship exists between corals and their symbiotic microorganisms, selecting for the coral holobiont that is best suited for the prevailing environmental conditions. Here, we discuss the microbial-host relationships within the coral holobiont, along with their potential roles in maintaining coral health. We propose the term BMC (Beneficial Microorganisms for Corals) to define (specific) symbionts that promote coral health. This term and concept are analogous to the term Plant Growth Promoting Rhizosphere (PGPR), which has been widely explored and manipulated in the agricultural industry for microorganisms that inhabit the rhizosphere and directly or indirectly promote plant growth and development through the production of regulatory signals, antibiotics and nutrients. Additionally, we propose and discuss the potential mechanisms of the effects of BMC on corals, suggesting strategies for the use of this knowledge to manipulate the microbiome, reversing dysbiosis to restore and protect coral reefs. This may include developing and using BMC consortia as environmental “probiotics” to improve coral resistance after bleaching events and/or the use of BMC with other strategies such as human-assisted acclimation/adaption to shifting environmental conditions.

## Introduction

Microorganisms are key components of all multi-cellular life due to their crucial roles in nutrient cycling and metabolism (Ainsworth et al., 2010; Garren and Azam, 2012; Thompson et al., 2014). In the past few decades, the important relationship between coral and its microbial symbionts have been documented (reviewed in Bourne et al., 2016). However, coral-microbiome interactions are not yet fully understood due to complex interactions, which include host modulation of the physiology of symbiotic cells (Barott et al., 2015) and the influence of symbiotic cells on their host (Sharp and Ritchie, 2012). The responses of coral-associated microbial communities to shifts in coral health (Harvell et al., 2007; Bourne et al., 2008; Jones et al., 2008; Glasl et al., 2016; Guest et al., 2016) and environmental stressors (Hughes et al., 2003; Doney et al., 2012; Vega Thurber et al., 2014; Garren et al., 2015; Kwiatkowski et al., 2015) have been extensively explored and reported. Changes in environmental conditions may modify coral physiology, leading to changes in the structure, spatial arrangement and abundance of the local population. These environmental disturbances can also directly or indirectly induce shifts in the associated microbial communities, leading to the emergence of disease (Harvell et al., 2007; Mouchka et al., 2010), which is an ongoing threat to coral reefs worldwide (Mumby and Steneck, 2008; Harvell et al., 2007; Vega Thurber et al., 2009; Krediet et al., 2013; Rogers and Miller, 2013).

Through shuffling of the dominant photosynthetic *Symbiodinium* clades within their tissues, some corals have become more tolerant to seawater temperature increases, thereby avoiding repeated bleaching events (Buddemeier and Fautin, 1993; Baker et al., 2004; Berkelmans and van Oppen, 2006; Abrego et al., 2008; Jones et al., 2008). However, corals may revert to the original clade (i.e., sensitive clade) over the long-term when the stress is removed (Thornhill et al., 2006). The coral bacterial communities are also sensitive to environmental changes and may also be involved in coral resilience (Reshef et al., 2006; Santos et al., 2014, 2015, 2016; Thompson et al., 2014). However, little is understood about the permanence of these shifts in the coral microbiome in the face of changing environmental conditions (Rowan et al., 1997; Thornhill et al., 2006; Thompson et al., 2014) and whether shifting microbial baselines can provide the resilience needed for corals facing mounting environmental stresses. Additionally, some reports have also indicated the importance of the coral genome to resilience (Barshis et al., 2013; Bhattacharya et al., 2016; Howells et al., 2016). Thus, there is still much to be explored about the relationship and the actual role of coral-associated microbiota—including *Symbiodinium*—in healthy conditions or when homeostasis breaks down.

**FIGURE 1:**
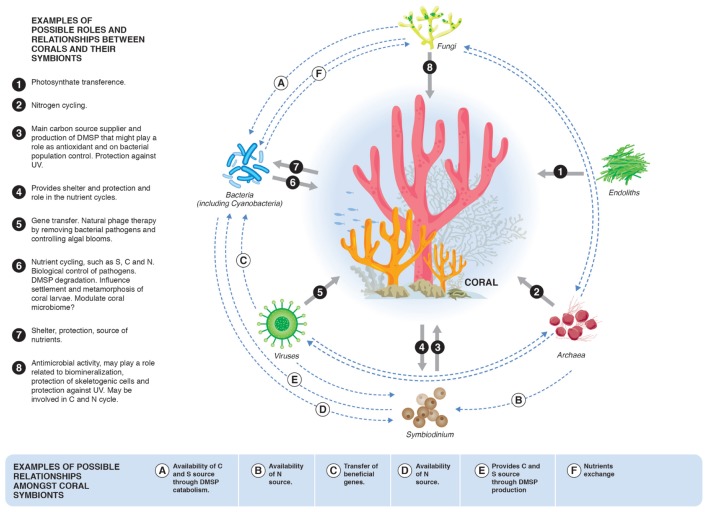
**Possible roles and relationships between corals and their symbionts and symbiotic microbial groups.** It is important to highlight that these mechanisms and interactions are some examples of potential BMC mechanisms. Other BMC roles still to be discovered are likely to be important targets in future investigations.

**FIGURE 2:**
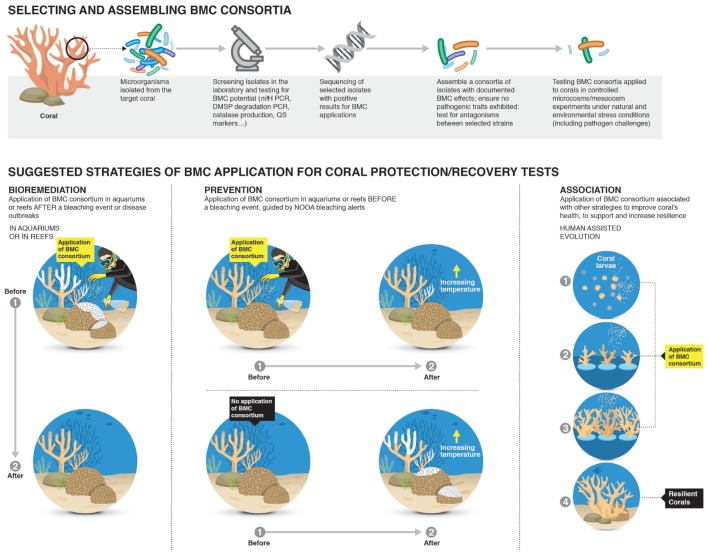
**Potential strategies for identifying and application of Beneficial Microorganisms for Corals (BMCs) for increasing coral resilience.** The first step would be to randomly isolate microorganisms from the surrounding reef water and the target coral species. Then the BMC would be identified and screened for beneficial interactions with the coral host through aquarium based experiments and the mechanisms by which the microorganisms confers benefits to the coral host identified. Extensive screening of BMCs would be undertaken to ensure no pathogenic interactions occurs and investigate potential antagonistic interactions between consortia of selected BMCs. The final steps would be application of the developed strategy in large mesocosm systems under relevant environmental stress conditions and including bacterial challenges to assess effectiveness of treatments before any field trials can begin.

The “Coral Probiotic Hypothesis” (CPH) (Reshef et al., 2006) postulates that the coral microbiome can be modulated to improve coral health and resilience. Little is currently known about the mechanisms related to the “probiotic” protection provided by the coral microbiome and whether these mechanisms can actually be considered “probiotics”. The objective of this review is to focus on the role of beneficial microorganisms associated with coral, including their identification, mechanisms of interaction with their host and their possible manipulation to improve coral fitness. We propose the term “Beneficial Microorganisms for Corals” (BMC) for these coral “probiotic” microorganisms, in analogy to the Plant Growth Promoting Rhizobacteria (PGPR) (Kloepper and Schroth, 1978), which are well-described symbionts of plants that possess specific mechanisms to promote plant growth and development (Podile and Kishore, 2007; Lugtenberg and Kamilova, 2009). This term directly refers to the symbiont microorganisms that are players in the maintenance and protection of the coral physiological balance. This comparison can be made if we define the BMC mechanisms that promote coral health and use current published examples that detail the mechanisms by which candidate BMC promotes coral health. The network of beneficial interactions provided by some symbiotic microorganisms is summarized in **Figure 1** and detailed further in subsequent discussion sections. Within plant sciences, a similar approach has been widely explored within the complex rhizo-microbiome environment, which is inhabited by a wide range of microorganisms. Some beneficial microbes in the rhizosphere improve plant health and promote growth through direct and/or indirect mechanisms such as enhancement of plant nutrition (e.g., nitrogen fixation, solubilization of phosphate or production of siderophores), biological control of plant pathogens and induction of plant defense systems, among others (Götz et al., 2006; Couillerot et al., 2009; do Carmo et al., 2011; Scheuring and Yu, 2012; Mendes et al., 2013; Vacheron et al., 2013). We propose to evaluate BMC in the same way, assembling the beneficial mechanisms of individual microorganisms to generate a cluster of targets and search for microbial groups that should be better understood and perhaps manipulated to improve coral resilience (see **Figure 2**). The selection and application of potential BMC can be achieved by (i), isolating microbial organisms that have potential BMC roles; and (ii), assembling and testing the ability of these BMC to convey resilience to corals subject to environmental stress and biotic and abiotic challenges (both experimentally and *in situ*). Since the coral microbiome is potentially a key factor affecting coral resilience, its manipulation is one action that can be developed to protect and preserve coral reefs.

## Applying the Probiotic Concept to Corals

Discussion of the term “probiotic” is important to evaluate and determine its applicability to BMC. While there are several definitions of the term “probiotic,” it is widely used in the context of “a live microbial feed supplement that beneficially affects the host animal by improving its intestinal balance” (Fuller, 1989). It was first created to refer to gram-positive bacteria associated with the genus *Lactobacillus* and its effects in mammalian hosts (Fuller, 1989; Verschuere et al., 2000). The definition provided by the Food and Agriculture Organization of the United Nations and the World Health Organization (FAO/WHO, 2001/2002) is “live microorganisms that, when administered in adequate amounts, confer a health benefit to the host.” When proposing the CPH, Reshef et al. (2006) asserted that the term “probiotic” could also be applied to invertebrates such as corals. Although corals and other marine invertebrates do not themselves possess an intestine, they harbor diverse microorganisms that assist in the maintenance of their fitness (Krediet et al., 2013; Thompson et al., 2014). The use of the term “probiotic” to simply describe microorganisms that can provide benefits to the host (pro – “in favor of something” and “biotic” – biological) or their ecosystems would be a natural adaptation of this term. However, we must also consider that the most accurate meaning of the term “probiotic” is restricted and encompasses features that are needed to determine probiotic assignment. The International Scientific Association for Probiotics and Prebiotics (ISAPP) recognizes an overall framework for the use of the term “probiotic” (Hill et al., 2014) and discusses the regulation of these products, which is not trivial. Furthermore, coral symbionts can provide many advantages to corals other than nutritional benefits, including biological control of pathogens. PGPR provide similar beneficial features in plants (Kloepper and Schroth, 1978), which are widely used to improve agricultural sustainability and productivity (Peixoto et al., 2010; Singh et al., 2011; Bhardwaj et al., 2014). The CPH is an important and acceptable explanation of the intrinsic relationship within the holobiont. Although the use of the term “probiotic” is not wrong as a natural adaptation of the terminology, and considering the international requirements for classifying probiotic products (Hill et al., 2014), we herein propose the use of the term BMC to specifically address coral symbionts that possess potential beneficial traits, including nutritional (“probiotics”) and protective mechanisms that improve coral fitness and contribute to coral resilience (see **Table 1** and **Figure 1**).

**Table 1:** Examples of proposed BMC (beneficial microorganisms for corals) characteristics and potential beneficial mechanisms.

Proposed Beneficial Characteristic (BMC characteristics)	Beneficial mechanism	Examples of references describing the beneficial mechanism
Photosynthesis	Input of organic compounds to the holobiont	Verbruggen and Tribollet, 2011; Burriesci et al., 2012; Davy et al., 2012; Tremblay et al., 2012
Nitrogen fixation	Input of fixed nitrogen to the holobiont	Olson et al., 2009; Lema et al., 2012; Santos et al., 2014; Bednarz et al., 2015; Cardini et al., 2015
Fixed nitrogen and carbon cycling and regulation	Control of organic compound distribution	Kimes et al., 2010
Production of dimethylsulfoniopropionate (DMSP)	Bacterial populations control on the coral surface	Barott and Rohwer, 2012
Degradation of dimethylsulfoniopropionate (DMSP)	Increase carbon and sulfur availability; production of sulfur-based antimicrobial compounds such as tropodithietic acid (TDA)	Kirkwood et al., 2010; Raina et al., 2016
Production of mediated signals to larval settlement facilitation	Contribute to larval settlement modulation or regulation	Webster et al., 2004; Heyward and Negri, 2010; Ritson-Williams et al., 2010; Shikuma et al., 2014
Production of antibiotics and competition with pathogens	Biological control of pathogens	Ritchie, 2006; Gochfeld and Aeby, 2008; Kirkwood et al., 2010; Alagely et al., 2011; Kvennefors et al., 2012
Production of quorum sensing (QS) signal molecules, such as *N*-acylhomoserine lactones (AHLs)	Allow microbial interactions within the holobiont; can act on bacterial colonization control, bioluminescence, pathogenesis control and extracellular enzyme production	Henke and Bassler, 2004; Ng and Bassler, 2009; Tait et al., 2010; Sharp and Ritchie, 2012; Certner and Vollmer, 2015; Meyer et al., 2015
Mechanisms influencing the protection of skeletogenic cells	Enhance the survival of skeletogenic cell types	Domart-Coulon et al., 2004
Production of mycosporine-like amino acids (MAA)	Protection of coral tissue against ultraviolet radiation	Dunlap and Shick, 1998

## BMC Mechanisms

The coral holobiont comprises the coral host and its associated microorganisms, including *Symbiodinium*, bacteria, viruses, archaea, fungi, endolithic algae and protists (Rohwer et al., 2002; Rosenberg et al., 2007). Distinct microbial communities can colonize various coral microhabitats, such as the surface mucopolysaccharide layer (SML), coral tissue, gastrovascular cavity and coral skeleton (Rosenberg et al., 2007; Agostini et al., 2012; Glasl et al., 2016). A number of other organisms, including fishes, crabs, and a range of crustaceans, bivalves and worms also belong to the group of eukaryotes associated with coral tissue and its calcareous skeleton (Rosenberg et al., 2007; Bourne et al., 2009; Ainsworth et al., 2010; Janouškovec et al., 2013; Šlapeta and Linares, 2013). Although the diversity of organisms that interact with the coral host is highly dynamic and often poorly described, the wide variety in the coral-associated microbiome enhances the range of potential BMC functions that these microorganisms can play in the holobiont.

The coral holobiont concept provides a framework to discuss how the associations among the different groups and shifts in these associations can influence coral health and the holobiont symbiotic community (Bourne et al., 2016). Coral-associated microorganisms have important roles in maintaining dynamic holobiont homeostasis, forming a network of connections that include carbon uptake, nitrogen and sulfur cycling and production of antimicrobial agents, thereby facilitating biological control of pathogens (Ainsworth et al., 2010; Krediet et al., 2013; Raina et al., 2016) (**Table 1** and **Figure 1**). In addition, bacterial biofilms on reef substrata can serve as cues to facilitate settlement of coral larvae (Hadfield, 2011). Coral-associated fungi can also protect coral tissue against ultraviolet radiation through the production of protective molecules such as mycosporine-like amino acids (MAA) (Dunlap and Shick, 1998) and can enhance the survival of skeletogenic cell types (Domart-Coulon et al., 2004) (**Figure 1**). **Table 1** identifies some of the most promising candidate BMCs and their interactions within the coral holobiont, which can potentially be modulated to facilitate improved coral fitness and resilience to environmental shifts.

### Carbon Cycling

Photosynthesis, the process of producing fixed carbon from carbon dioxide and water using light-derived energy, is one of the most important known BMC mechanisms provided by the endosymbiotic *Symbiodinium* present within the cnidarian’s gastrodermis cells (Wakefield et al., 2000) (**Table 1** and **Figure 1**). *Symbiodinium* are highly efficient in their use of solar energy (Brodersen et al., 2014), producing organic compounds that contribute significantly to coral nutrition. Approximately 60–80% of *Symbiodinium* photosynthetic fixed carbon is transferred to the coral host (Tremblay et al., 2012), primarily as glucose (Burriesci et al., 2012). This glucose is used for coral growth, reproduction, respiration and biocalcification (Davy et al., 2012). A variety of *Symbiodinium* strains have been identified and are distributed into 9 clades (A–I) (Pochon et al., 2006; Pochon and Gates, 2010; Blackall et al., 2015). However, only 6 have been identified in corals: A, B, C (Rowan and Powers, 1991), D (Baker, 2003), F (LaJeunesse, 2001) and G (Van Oppen et al., 2005). Type D symbionts are known to be the most heat tolerant (Silverstein et al., 2017), and as such, corals subject to repeated bleaching events have a higher proportion of this symbiont (Baker et al., 2004; Berkelmans and van Oppen, 2006; Stat and Gates, 2011; Lesser et al., 2013; Silverstein et al., 2017). Recent data from a rare *Symbiodinium* biosphere have demonstrated the *de novo* acquisition of *Symbiodinium* types from surrounding water by adult corals and indicate that an important switching strategy may contribute to holobiont thermal tolerance (Boulotte et al., 2016). It is known that corals in some of the hottest seas of the world have developed a symbiotic relationship with algae, which facilitates thermal tolerance to the host (Howells et al., 2013; Hume et al., 2015). The genomic blue-print of *Symbiodinium* is beginning to be unveiled; comparative analyses demonstrate that all dinoflagellates have significantly more transmembrane transporters, especially those associated with carbon and nitrogen delivery, compared to other eukaryotes (Aranda et al., 2016). Species-specific expansions in these transporters can potentially provide a genomic explanation for specific *Symbiodinium* clade adaptations to different hosts and environments. Increasing genomic information for all BMCs, including *Symbiodinium*, will help to identify traits essential for symbiosis and the shared functional capacity that is critical for a stable coral holobiont.

Manipulation of *Symbiodinium* populations *in hospite*, i.e., inoculating more thermally tolerant strains (as part of BMC consortia, as described in **Figure 2**), is one mechanism to increase the resilience of the coral holobiont to thermal stress (Berkelmans and van Oppen, 2006; van Oppen et al., 2015). *Symbiodinium* are often manipulated in nature itself; corals can naturally shuffle *Symbiodinium* populations with some being more heat tolerant (Buddemeier and Fautin, 1993; Baker et al., 2004, 2016; Berkelmans and van Oppen, 2006; Abrego et al., 2008; Jones et al., 2008). However, the long-term stability of manipulated coral/*Symbiodinium* associations is still unclear and requires further investigation (Thornhill et al., 2006; Baker et al., 2016). Efforts centered on coral propagation and reef restoration are increasing globally (Young et al., 2012), in response to alarming declines in coral reefs in some regions of the world (Hoegh-Guldberg et al., 2008; De’ath et al., 2012). These efforts offer the opportunity to test the manipulation of *Symbiodinium* strains through inoculation of early life stages of corals with resilient clades that offer the best hope for growth and survival in restoration areas. However, the trade-offs in coral life history traits must be fully explored to ensure that such manipulations do not solve one problem and introduce another into the coral population.

Many current studies are focused on identifying the prokaryotes associated with corals and, more importantly, elucidating their function, including pathways for nutrient sharing and passage within the holobiont (reviewed in Bourne et al., 2016). This ability of microbes to metabolize nutrients, which can then be translocated to their host, is likely a driver in the establishment of coral-associated microbial assemblages. Coral metagenomic studies are beginning to identify several genes in the central carbon metabolism pathways, including carbon fixation and degradation genes (Kimes et al., 2010). However, the presence of a functional gene does not necessarily imply functionality, and further *in situ* research or metatranscriptomic and metabolomic analyses are needed to improve our knowledge of the role of the microorganisms in driving nutrient cycling in corals.

Williams et al. (2015) have suggested a potential, and important, role of the endolithic algae influencing the bacterial diversity within the coral tissue. Endolithic microbial communities are often a forgotten component of the coral holobiont (Yang et al., 2016). These organisms have also a potential active role in protecting corals during bleaching events through basal photosynthetic activity, translocating nutrients from the skeleton to the coral tissue and keeping the coral alive until re-colonization of *Symbiodinium* (Fine and Loya, 2002; Verbruggen and Tribollet, 2011). A greater understanding of the function of all prokaryote communities and their niche location within the holobiont is essential to defining the BMC and then elucidating mechanisms to manipulate them for the benefit of corals. For instance, the use of network analysis, evaluating negative and positive interactions between microorganisms and their roles, has been suggested as moving the field of microbial ecology in corals forward (Sweet and Bulling, 2017) and would be extremely useful to elucidate such mechanisms.

Within the coral holobiont, symbiosis can occur not only between the coral host and its photosymbionts or between the coral host and its microbiome but also as a mutualistic relationship between the photosymbionts and the microbiome. The growth and density of dinoflagellate populations within the coral host are highly dependent on available nutrients. As such, the microorganisms mediating nutrient cycling likely have an important role in the stability of the *Symbiodinium* population and consequently aid in the maintenance of coral-symbiotic algae interactions (Santos et al., 2014; Rädecker et al., 2015). This indicates that BMC mechanisms are not only specific to the coral host but could also be represented by beneficial interactions between coral microbial symbionts. Any manipulation must account for these complementary interactions within the holobiont. Therefore, the use of consortia (as opposed to single-strain inoculation) with a range of mechanisms for beneficial outcomes for the coral is strongly recommended. However, evaluating the potential benefits and/or detrimental roles that each BMC has in a consortium, rather than individually, is challenging and requires detailed investigation in model systems.

### Other Essential Nutrient Cycling Pathways

Metagenomic studies have identified the presence of genes involved in nitrogen cycling pathways *via* nitrogen fixation, ammonification, nitrification, and denitrification within the coral holobiont (Wegley et al., 2007). Ubiquitous nitrogen-fixing bacteria have been reported in corals as inferred by recovery of nitrogenase (*nifH)* gene diversity, with dominant taxa representing the α-, β-, γ-, and δ-proteobacterial classes (Olson et al., 2009; Lema et al., 2012; Santos et al., 2014). The nitrogen provided by this process is likely to support the host and its associated microbiota, including *Symbiodinium* (Santos et al., 2014). It is estimated that diazotrophs provide up to 11% of *Symbiodinium* nitrogen requirements (Cardini et al., 2015). Lesser et al. (2007) demonstrated that *Symbiodinium* cells associated with *Montastrea cavernosa* acquire N from cyanobacterial endosymbiotic diazotrophs; this ability seems to increase with depth and is dependent on heterotrophy. In addition, some fungal species have been hypothesized to be involved in nitrogen metabolism through conversion of nitrate and nitrite to ammonia, thereby enabling fixed nitrogen to cycle within the coral holobiont (Wegley et al., 2007). Archaea may also be central to nitrogen recycling within corals, likely through nitrification and denitrification processes, and thus regulate ammonium concentrations (Siboni et al., 2012).

Members of the Roseobacteriales group (also involved in sulfur cycling) are often identified as obligate associates within *Symbiodinium* cultures, increasing the growth rate of dinoflagellates (Ritchie, 2011). Bacteria/algae interactions can be affected under environmental disturbance and, in turn, can affect the holobiont as a whole. Exploring and understanding these interactions will facilitate the development of methodologies to manipulate the nitrogen-fixing microbiome, stimulating specific groups by adding nutrients or inoculating key BMC groups (BMC consortia) to increase or regulate nitrogen inputs (**Figure 2**).

*Symbiodinium* and several bacterial groups have a central BMC role within the sulfur cycling pathways of the coral holobiont (**Table 1** and **Figure 1**). The *Symbiodinium* are large producers of dimethyl sulfate compounds (DSCs), which take part in the antioxidant system of corals (Deschaseaux et al., 2014) and also potentially in structuring coral-associated bacterial communities that cycle carbon and sulfur within the holobiont (Raina et al., 2009, 2010). Coral-associated bacterial groups, including members of the *Flavobacteriaceae* (Howard et al., 2011) *Halomonas* (Todd et al., 2010), *Roseobacter, Pseudomonas*, and *Oceanospirillales* (Raina et al., 2010, 2013), are capable of metabolizing dimethylsulfoniopropionate (DMSP) and consuming its products for their own metabolic processes. The catabolism of DMSP also potentially generates sulfur-based antimicrobial compounds such as tropodithietic acid (TDA), which at low concentrations (0.5 μg/mL) can inhibit the growth of the coral pathogens *Vibrio coralliilyticus* and *V. owensii* (Raina et al., 2016). Thus, the production and metabolism of sulfur compounds represents a potential BMC mechanism, and the manipulation of these key microbial groups may promote coral health through the regulation of key symbiotic populations, antimicrobial activity and nutrient input.

### Production of Antibiotics and Competition With Pathogens

Recent studies have focused on the biological control promoted by native bacteria in the regulation of bacterial colonization on the coral surface, which consequently controls resistance against diseases (Bourne et al., 2016; Egan and Gardiner, 2016; Glasl et al., 2016). Corals can protect themselves against pathogen infection using the mucus microbiome as a barrier against potential pathogens (Glasl et al., 2016). Some protective mechanisms include competition for nutrients and/or space, and/or production of antibiotics in mucus (Ritchie, 2006) or coral tissue (Gochfeld and Aeby, 2008). Shifts in the native microbial community may have a negative impact on the host, i.e., dysbiosis (Petersen and Round, 2014), leading to the onset of disease and eventual mortality (Egan and Gardiner, 2016). For instance, thermal stress can induce changes in coral mucus and a consequent shift in the microbiome, which in turn could influence holobiont homeostasis (Lee et al., 2016).

Several studies have demonstrated that bacteria isolated from corals are able to inhibit the colonization and growth of many other types of bacteria through antibacterial activity, including putative pathogens of coral such as *Vibrio shiloi* (Rypien et al., 2010), *V. coralliilyticus* (Kvennefors et al., 2012) and *Serratia marcescens* (Alagely et al., 2011). Thus, production of antibiotics and niche competition with pathogens are strong BMC mechanisms provided by the coral microbiome (**Table 1**). The biological control of bacteria, including pathogens, can also be performed by viruses and protists (**Figure 1**), which are the two dominant top–down control mechanisms of bacteria in the open ocean (Chow et al., 2014). Recent studies have indicated the important role of viruses in coral fitness, either causing diseases or promoting coral health (reviewed in Sweet and Bythell, 2016 and Vega-Thurber et al., 2017). Corals can harbor a great diversity of bacteriophages and archaeal phages, feasibly involved in key ecological interactions and genetic material exchange (Sweet and Bythell, 2016), playing a crucial role in the reef microbial dynamics and biogeochemical cycling (Vega-Thurber et al., 2017). However, despite their role in the biological control of specific bacteria, Chow et al. (2014) correlated shifts in microbial structures and detected positive interactions between bacteria and viruses, suggesting that viruses may not only control but also follow their host. This may indicate that viruses can control pathogens and be used in association with BMC consortia. Successful phage therapy experiments developed in small aquarium demonstrate the prevention of the progression of bacterially mediated lesions on infected corals (Efrony et al., 2009; Cohen et al., 2013). Therefore, the effects of viruses on bacterial development and competitiveness should be analyzed as a potential factor to be manipulated for enhancing BMC effectiveness.

Some bacterial groups, such as *Endozoicomonas*, are predominant in healthy corals, but the relative abundance of this group decreases in compromised or diseased corals (Bayer et al., 2013; Vezzulli et al., 2013; Meyer et al., 2014; Glasl et al., 2016; Neave et al., 2016; Morrow et al., 2017). The genus *Endozoicomonas* belongs to the family *Hahellaceae* and the order *Oceanospirillales*, a group of heterotrophic aerobic marine bacteria that were first described by Kurahashi and Yokota (2007). Although the functional role of *Endozoicomonas* is not well understood, this genus has been described as a very diverse and flexible symbiotic group (Neave et al., 2017) that is associated with several marine hosts and is globally distributed. The genus occurs in sponges, fishes, corals and others (Kurahashi and Yokota, 2007; Bourne et al., 2008; Choi et al., 2010; Jensen et al., 2010; Yang et al., 2010; Speck and Donachie, 2012; Pike et al., 2013). Additionally, some *Endozoicomonas* strains have been identified as producers of antimicrobial compounds (Ritchie, 2006; Rua et al., 2014) and may have a role in sulfur cycling (Raina et al., 2009). Ainsworth et al. (2015) detected members of the order *Endozoicimonaceae* in the coral mucus and/or skeleton, though this group was not part of the symbiotic core microbiome associated with the coral species *Montipora capitate, Acropora granulosa*, and *Leptoseris* spp. Altogether, these findings highlight the potential role of this group in the biological control of coral pathogens. Recent genomic studies indicate that *Endozoicomonas* may be able to recognize, translocate, communicate and modulate the coral host (Ding et al., 2016) plus contribute to protein provision and cycling of carbohydrates (Neave et al., 2017). Neave et al. (2017) demonstrated evidence for specific symbiotic mechanisms for different *Endozoicomonas* ecotypes associated with different coral hosts though these ecotypes likely have a non-symbiotic life stage due to possession of large genomes which have not been narrowed for obligate endosymbiosis. These studies highlight the importance of *Endozoicomonas* for corals and other marine hosts in potentially developing one or more BMC mechanisms.

### Quorum Sensing

Little is known about quorum sensing (QS), a system of bacterial cell-cell chemical signaling, within the coral holobiont. It is necessary to confirm the production of QS signals by coral-associated commensals and pathogens under laboratory conditions as well as to detect them in natural environments. However, evidence from other microbial-host interactions indicates that QS can be a beneficial mechanism to improve coral health and resilience through the control of native and/or pathogenic populations. For example, QS systems are important for PGPRs, specifically legume-nodulating rhizobial nitrogen-fixing symbiotic cells, which are strongly influenced by QS signaling and often control other bacterial populations (Wisniewski-Dyé and Downie, 2002; Chin-A-Woeng et al., 2003; Gonzalez and Marketon, 2003; McAnulla et al., 2007; Sanchez-Contreras et al., 2007). QS genes are also involved in several bacterial physiological adaptations (e.g., light and antibiotic production), allowing bacteria to change their behavior and improve niche competitiveness (reviewed in Sanchez-Contreras et al., 2007).

Quorum sensing may be one mechanism used to modulated bacterial–host interactions at the coral surface (Sharp and Ritchie, 2012). Coral mucus/microorganism interactions are likely competitive, with dominant communities potentially secreting QS disruptive compounds that influence the colonization, bioluminescence, pathogenesis and extracellular enzyme production in a number of bacterial species, including some from the genus *Vibrio* (Ng and Bassler, 2009). For example, *Vibrio* growth dynamics and competitiveness in coral mucus and tissues has been demonstrated to be linked to QS signaling molecules such as *N*-acylhomoserine lactones (AHLs) (Henke and Bassler, 2004; Tait et al., 2010; Certner and Vollmer, 2015). These *Vibrio-*derived QS molecules are also influenced by environmental factors such as temperature (Tait et al., 2010; Saha et al., 2015) with QS mechanisms breaking down under stress conditions that disrupt the associated microbiome. Certner and Vollmer (2015) demonstrated that resident microorganisms can opportunistically cause white band disease in *Acropora cervicornis* and that this seems to be regulated by a quorum-sensing signaling molecule. Manipulating QS mechanisms to promote beneficial microbes over opportunistic or pathogenic microbes is one potential strategy to improve coral fitness during times of stress (**Table 1**). However, it is necessary to increase our knowledge of these interactions to establish a successful manipulation process.

## Core Microbiome: A Useful Coral Health Indicator

Defining the stable and ubiquitous core microbiome, the variable microbial species associated with coral (and responsive to environmental conditions) and the spatially and/or regionally microbial core (niche-specific) (Hernandez-Agreda et al., 2016; Sweet and Bulling, 2017) can inform the health status of coral relative to the environment and constitutes a viable approach to identify potential BMCs. The core microbiome is composed of common members, host-specific, of two or more microbial communities (Turnbaugh et al., 2007; Hamady and Knight, 2009; Hernandez-Agreda et al., 2016; Sweet and Bulling, 2017). Studies have suggested that identification of the operational taxonomic units (OTUs) that compose the core microbiome is vital because these populations may play key roles (potential BMC) due to their ability to maintain stability in the face of environmental changes (Shade and Handelsman, 2012; Shafquat et al., 2014; Ainsworth et al., 2015; Chu and Vollmer, 2016). This approach has been applied to identify the interactions between other hosts and microorganisms, including mammalian intestines and plant roots (Shade and Handelsman, 2012; Shafquat et al., 2014). Identification of the central metabolic pathways associated with the core microbiome will also provide vital information about how host–microbiome interactions are established and maintained (Shafquat et al., 2014; Ainsworth et al., 2015). The “functional coral core microbiome” is potentially more important than a taxonomic core, as demonstrated for seaweeds, with functional redundancy widely observed in complex microbial communities (Burke et al., 2011). Therefore, while microbial community diversity may change in response to environmental conditions, essential functions can be maintained by the new taxonomic groups. Importantly, the core microbiome is commonly associated with host-constructed niches, and these microorganisms are therefore less sensitive to the surrounding environment (Hester et al., 2015). However, they are also potentially capable of adapting to environmental change (McFall-Ngai et al., 2013; Santos et al., 2014).

Ainsworth et al. (2015) identified the bacterial symbionts that compose the core microbiome in the corals *Acropora granulosa, Montipora capitata*, and *Leptoseris* spp. The core microbiome in various coral niches were identified, including bacteria specific to the gastrodermis cells and symbiotic *Symbiodinium* dinoflagellates (Ainsworth et al., 2015). Chu and Vollmer (2016) detected a stable bacterial community (bacteriome core) associated with six coral species from the Caribbean region, suggesting that the host is the stronger driver of this core and also indicating specific and divergent niches for bacteria. While there is an identifiable core coral microbiome, there is also a dynamic microbiome that varies depending on species, season, habitat and life stage and is likely a product of stochastic events or a response to changing environmental conditions (Hester et al., 2015). This dynamic community is also an important target for BMC studies because it can represent a source of strains that are adaptive to specific conditions of environmental stress. Acquisition and/or shifts in microorganisms have been suggested as an important tool for coral adaptation and are potentially one mechanism for increasing resilience under varied environmental conditions (Zilber-Rosenberg and Rosenberg, 2008; Singh et al., 2013; Ziegler et al., 2017).

Beneficial Microorganisms for Corals should be identified and potentially manipulated for each individual coral species, each regional location and each stage of development. For example, in the case of humans, a doctor will always provide individual analysis, considering specific medical records pertaining to a specific patient, including their age, prior to prescribing a medication or treatment. Likewise, a microbial consortium, specifically isolated from and developed for oil bioremediation in polar areas, may not be the best option for oil bioremediation in tropical areas because strains may not be well adapted to a new environment with a range of different biotic and abiotic interactions. Environmental recovery and protection strategies can be applied to various habitats, provided that they are subject to the local conditions of that environment. It is important to follow strict ethical guidelines, as currently indicated for microbiome studies and manipulation of other organisms (Rhodes et al., 2013), to avoid undesirable side effects. The manipulation of native and non-modified microorganisms is the first step to achieve this.

### Coral Diseases, a Disruption Within the BMCs?

Coral diseases may occur in response to biotic stresses caused by bacteria, fungi, and viruses (Davy et al., 2006; Bourne et al., 2009; Krediet et al., 2013) and/or abiotic stresses, such as rising temperature, ultraviolet radiation, sedimentation and pollution (Harvell et al., 2002, 2007; Bruno et al., 2003; Burge et al., 2014). The disruption of the BMC community (both core and dynamic microbial communities) is likely an important trigger for disease establishment. The term “disease” has a variety of definitions and essentially describes a shift away from a healthy state that may be caused by exogenous (external, environmental) and endogenous (internal, from the organism itself) factors (Scully, 2004). In recent years, coral diseases have emerged as a significant threat to reefs around the world. From the time the first coral disease was described in Antonius (1973) to today, more than 30 diseases have been reported (Green and Bruckner, 2000), which demands an urgency in understanding coral–microbiome interactions. In general, these diseases are identified by changes in coral coloration, whose characteristics provide the names of the diseases, such as black band, white band, yellow band, and white pox, among others. Coral diseases have the potential to cause widespread mortality and thereby significantly change the structure of reef communities (Porter et al., 2001; Sutherland and Ritchie, 2004; Miller et al., 2009). However, little is known about the causes and effects of these diseases, including the etiology, transmission route, prevention, control and reduction of their impacts. Disruption of homeostasis may result in physiological changes that may cause disease or even lead to the mortality of the whole colony. It is possible that this disruption is caused by an imbalance in BMC mechanisms, or dysbiosis, i.e., loss of antimicrobials and loss of nutritional pathways, which then leads to disease. The mechanisms of disruption could be unveiled by BMC studies, where the absence of benefits can be regarded as potential disease mechanisms. For instance, it is suggested that *Symbiodinium* cells require nitrogen that is provided by coral-associated nitrogen-fixing bacteria (Cardini et al., 2015). However, environmental thermal disturbances lead to changes in the abundance and diversity of these nitrogen-fixing communities (Santos et al., 2014). Initially, the holobiont can adapt to supply *Symbiodinium* and consequently meet coral requirements. However, if a thermal disturbance persists, it is likely that a breakdown in the homeostasis may occur. Knowledge of BMC mechanisms and symbiotic relationships within coral could potentially aid in the development of microbiome management strategies that may avoid any such disruption in coral/microbial associations (**Figure 2**) and reverse dysbiosis. For instance, specific nitrogen-fixing bacteria could be inoculated during events of thermal stress. This procedure could delay the effects of environmental impact within the holobiont, which would then be capable of withstanding environmental stress until the restoration of optimal environmental conditions. It is important to understand the crucial breaking point when the symbiotic interaction fails; fine-scale and dynamic observation of both host and symbiont function will be required to do so. For instance, the phylogenetic and functional response of sponge holobionts to thermal stress was thoroughly described by Fan et al. (2013). Such comprehensive surveys are needed to fully understand what and when imbalances occur and pass a point of no return.

## Potential Manipulation of BMCs

The recovery and selection of potential BMC could be developed through basic microbiology methods using tailored culture media and cultivation strategies and screening for specific mechanisms (**Figure 1**). The selection of culture media can be targeted to our current understanding of the nutritional requirements of potential BMC organisms. BMC can be inoculated at different coral life stages, such as larval and or juvenile stages, prior to acquisition of *Symbiodinium* using similar approaches to those performed in agriculture through the inoculation of seeds with PGPRs (Götz et al., 2006). BMC inoculation could also be performed on adult corals with potentially novel applications, such as using microencapsulation and nanoparticles to heterotrophically feed adult corals and thereby transfer a BMC cocktail directly into the coelenteron. Use of knowledge derived from other systems will be essential to develop inoculation strategies suitable for corals. For example, microbial saline suspension and microbial immobilization of substrates such as biodegradable polymers are currently used in terrestrial environments (Ahmad et al., 2011) and aquaculture systems (Martínez Cruz et al., 2012). The use of cost-effectiveness and biodegradable substrates, such as the widely used alginate (Sivakumar et al., 2014), could potentially represent an environmentally friendly approach to encapsulation of BMCs and delivery to corals as heterotrophic feed particles, essentially similar to probiotics in humans.

Microbiome manipulation to enhance coral health could be used in association with other approaches for better results (**Figure 2**). For instance, human-assisted evolution (HAE) of coral involves the genetic natural enhancement of corals to improve their tolerance to stress (van Oppen et al., 2015). Some techniques may be used to naturally accelerate coral adaptation against these stresses, such as random mutations, natural selection, acclimatization and random changes in microbial symbiont communities (van Oppen et al., 2015). The human-assisted evolution of corals could be developed in association with the use of specific BMC consortia inoculants for specific coral species and environmental conditions, in addition to inducing the modulation of naturally generated microbiota in corals. BMC could be continually used during various stages of coral development (**Figure 2**), to thereby increase the coral survival rate. The use of phage therapy associated with BMC is also a promising approach to be tested, as BMC could improve coral fitness and phage could act to directly control potential pathogens.

The host–microbial interaction data provided by these experiments could be used to indicate functional microbiome dynamics at different coral life stages. Induced shifts in the microbiome of sensitive or diseased corals through the use of BMC consortia inoculants or modulated BMC populations are plausible options and could be considered an epigenetic process of acclimation (Heard and Martienssen, 2014). Furthermore, experiments that breed corals, maintain offspring under future climate scenarios (i.e., elevated temperature) and evaluate native microbiome responses under thermal stress conditions could provide important information about the contribution of BMC to coral resilience. Inoculation of various types of *Symbiodinium* at various temperatures and the analysis of the competitiveness of these different types, as well as the resilience potential of inoculated corals, would also provide important BMC mechanistic data that will be the basis of further manipulation. To evaluate the concept of protection transmission from resistant to sensitive corals, transplantation of the microbial community via inoculation of healthy macerated tissue from disease-resistant corals to sensitive ones (similar to fecal transplant) could also be tested. However, the concentrations of each of the protective populations may not be sufficiently high to support competitiveness of these beneficial introduced populations. It would also be interesting to encourage similar approaches extrapolating the term BMC to other marine hosts [i.e., Beneficial Microorganisms for Sponges (BMS), Beneficial Microorganisms for Seagrass (BMS), etc.] by manipulating and evaluating specific mechanisms related to varied marine hosts and evaluating the ecological data regarding marine host–microbiome interactions.

The selected and proposed mechanisms can, and should, be also expanded, as more information on the microbial beneficial traits for corals are described. There are several potential mechanisms to be explored to improve coral fitness. For instance, the production of reactive oxygen species (ROS) is a key response of marine organisms against environmental stress, such as thermal stress (Lesser, 2012). In the absence of a definite mechanism for coral bleaching, the most widely accepted model is that *Symbiodinium* sp. chloroplasts are heat-damaged by the light-induced generation of toxic ROS, which could also be associated with other cellular processes and pathways (Tolleter et al., 2013). Although several types of ROS have been described, with a gradient of reactivity and diffusivity across membranes (Lesser, 2006), the primary mechanism of ROS production in *Symbiodinium* seems to be through hydrogen peroxide formation at the end of the photosynthetic electron transport chain. Suggett et al. (2008) suggested that the thermal tolerance of *Symbiodinium* could be associated with adaptive restrictions related to photosynthesis and correlated sensitive phylotypes with higher H_2_O_2_ production. Increasing the *in hospite* concentration of catalase—an enzyme commonly found in organisms exposed to oxygen that catalyzes the decomposition of hydrogen peroxide to water and oxygen (Chelikani et al., 2004)— within the holobiont could minimize the concentration of ROS during periods of thermal stress. Exploiting the ability of specific coral-associated microorganisms to increase catalase production represents a BMC target to be explored as a potential BMC mechanism and mitigation strategy to minimize and buffer against severe bleaching episodes. Considering that there are other enzymes that harvest ROS, such as super oxide dismutase and lysozyme, several other mechanisms can be suggested and evaluated to minimize ROS concentration within corals under thermal stress, as research focusing on coral microbiome manipulation start to be developed. Likewise, several other groups and mechanisms should be better explored as potential BMC, including groups, such as cyanobacteria and endolithic algae. Research focusing on coral microbiome manipulation is beginning to be developed.

## Challenges and Concerns

Studies of microbiome manipulations have, to date, mostly focused on terrestrial hosts (Alivisatos et al., 2015) such as biological control and PGPR inoculants for agricultural purposes (Costa et al., 2006; Götz et al., 2006; Peixoto et al., 2006, 2010; Aboim et al., 2008; Berg, 2009; Rachid et al., 2013), human fecal transplants (van Nood et al., 2013) and probiotics for humans (Leite et al., 2013, 2015). Bioaugmentation or microbiome manipulation approaches are also very useful for the bioremediation of contaminated sites (Santos et al., 2011; Cury et al., 2015; Jesus et al., 2015). Even within these well-defined systems, microbiome manipulation is challenging due to the evolving and complex nature of host/microorganism interactions.

It is also important to highlight that BMC and the strategies for their application, must be tested in well controlled, realistic experimental systems before field application. Before initiating reef recovery, it is best to perform a survey of the target reef to determine which components would be more suitable to improve the health of the site. Factors such as the stability of the host/microbial associated community, the microbiome transmission route (i.e., vertical vs. horizontal) and the cross-species relationships (neutral, beneficial or pathogenic) must be established. For instance, it is important to ensure that selected BMC microorganisms are not members of the “Pathobiome”; a term recently suggested by Sweet and Bulling (2017) identifying the pathogenic members of the microbiome. As highlighted by Sweet and Bulling (2017), it is essential to understand the pathobiome members and the mechanisms of interaction within the holobiont. This concept is complementary to the BMC concept, as both aim to better understand the mechanisms of dysbiosis and the triggers of microbiome disruption within the holobiont. Strict and robust testing of BMCs must be performed in small scale experimental systems and scaled up to mesocosms, which mimic field conditions, before field application. Questions such as *in situ* microorganism growth characteristics, effectiveness, competitiveness (interactions between host and inoculated BMC), trade-offs, repeatability and mass application (ecosystem scale) all need to be considered and evaluated. Although potentially efficient, microbial manipulation is very specific, and specific studies and strategies must be applied for each site.

## Conclusion and Perspectives About BMC

Coral microbiologists continue to describe the potential mechanisms by which corals benefit from associations with microbial partners (**Table 1**), and the application of “omics”-based approaches will further provide important information about the BMCs associated with corals. We propose that microorganisms possessing at least one of the listed characteristics detailed in **Table 1** could be classified as a BMC and manipulated to assess potential effect on coral fitness and resilience. This approach requires robust assessment of the efficiency and safety, however, in laboratory conditions before field application, similar to what is undertaken in agriculture, bioremediation and probiotic use by humans. These approaches will be challenging and there are many large knowledge gaps that need to be filled before BMCs can be suitable to the real world. However, the first step is to encourage the discussion around the concept for corals and its possibilities for reef ecosystems. The second step is undertaking well designed laboratory based experimental manipulations of BMCs, evaluating the health outcomes for coral holobionts under different environmental conditions. Successful examples of microbial modulation in other organisms can guide these efforts to test BMCs. Considering the alarming declines reported for coral reefs globally (Hoegh-Guldberg et al., 2008) innovative solutions are required to halt or even reverse these trends. While manipulation of the microbiome to improve coral resilience may be challenging and even controversial, the necessity to preserve coral reefs is paramount and all options should be on the table. In fact, a “natural BMC manipulation” of the bacterial community (i.e., a positive correlation between bacterial community dynamics and coral heat tolerance), was recently suggested by Ziegler et al. (2017). At the very least, application of the BMC concept and manipulation of identified BMC candidates can improve our understanding of the vital role microbes play within the coral holobiont through integrative physiological, microbiological and ‘omic’ based approaches. Such detailed understanding is critical for corals and the reef ecosystems that they build, faced with on-going global declines. Coral “microbial-therapy” is a potential new area of study in the face of increasing threats to coral reefs that could have positive outcomes for reefs in the near future.

## Author Contributions

All authors listed, have made substantial, direct and intellectual contribution to the work, and approved it for publication.

## Conflict of Interest Statement

The authors declare that the research was conducted in the absence of any commercial or financial relationships that could be construed as a potential conflict of interest.
